# Using a Patient Portal to Increase Enrollment in a Newborn Screening Research Study: Observational Study

**DOI:** 10.2196/30941

**Published:** 2022-02-10

**Authors:** Lisa M Gehtland, Ryan S Paquin, Sara M Andrews, Adam M Lee, Angela Gwaltney, Martin Duparc, Emily R Pfaff, Donald B Bailey Jr

**Affiliations:** 1 RTI International Research Triangle Park, NC United States; 2 Department of Medicine University of North Carolina Chapel Hill Chapel Hill, NC United States

**Keywords:** electronic health records, patient portals, patient selection, research subject recruitment, race factors, racial disparities

## Abstract

**Background:**

Many research studies fail to enroll enough research participants. Patient-facing electronic health record applications, known as patient portals, may be used to send research invitations to eligible patients.

**Objective:**

The first aim was to determine if receipt of a patient portal research recruitment invitation was associated with enrollment in a large ongoing study of newborns (Early Check). The second aim was to determine if there were differences in opening the patient portal research recruitment invitation and study enrollment by race and ethnicity, age, or rural/urban home address.

**Methods:**

We used a computable phenotype and queried the health care system’s clinical data warehouse to identify women whose newborns would likely be eligible. Research recruitment invitations were sent through the women’s patient portals. We conducted logistic regressions to test whether women enrolled their newborns after receipt of a patient portal invitation and whether there were differences by race and ethnicity, age, and rural/urban home address.

**Results:**

Research recruitment invitations were sent to 4510 women not yet enrolled through their patient portals between November 22, 2019, through March 5, 2020. Among women who received a patient portal invitation, 3.6% (161/4510) enrolled their newborns within 27 days. The odds of enrolling among women who opened the invitation was nearly 9 times the odds of enrolling among women who did not open their invitation (SE 3.24, OR 8.86, 95% CI 4.33-18.13; *P*<.001). On average, it took 3.92 days for women to enroll their newborn in the study, with 64% (97/161) enrolling their newborn within 1 day of opening the invitation. There were disparities by race and urbanicity in enrollment in the study after receipt of a patient portal research invitation but not by age. Black women were less likely to enroll their newborns than White women (SE 0.09, OR 0.29, 95% CI 0.16-0.55; *P*<.001), and women in urban zip codes were more likely to enroll their newborns than women in rural zip codes (SE 0.97, OR 3.03, 95% CI 1.62-5.67; *P*=.001). Black women (SE 0.05, OR 0.67, 95% CI 0.57-0.78; *P*<.001) and Hispanic women (SE 0.07, OR 0.73, 95% CI 0.60-0.89; *P*=.002) were less likely to open the research invitation compared to White women.

**Conclusions:**

Patient portals are an effective way to recruit participants for research studies, but there are substantial racial and ethnic disparities and disparities by urban/rural status in the use of patient portals, the opening of a patient portal invitation, and enrollment in the study.

**Trial Registration:**

ClinicalTrials.gov NCT03655223; https://clinicaltrials.gov/ct2/show/NCT03655223

## Introduction

### Advent of Patient Portals and Their Use in Research Recruitment

Failure to recruit a sufficient number of participants is a common barrier to the successful and timely completion of research studies [1]. Insufficient accrual of participants may require additional resources to achieve target enrollment, and failure to meet enrollment goals may result in underpowered studies [2,3].

Electronic patient portals are web-based applications owned and administered by health care institutions that allow patients to access their electronic health records (EHRs). In the past two decades, a growing number of patients have used this technology to manage their health care and communicate with their providers [4-6]. Estimates of patient portal use vary by study, subpopulation, and measured outcome, but reports range from 25.8% to 84.1%, and longitudinal analyses indicate that utilization rates are growing [7-16]. In a national US sample, Turner et al [17] found that 24.9% of participants reported using at least one patient portal tool in 2017, compared to only 12.6% in 2011. As adoption becomes widely accepted, researchers have recognized the opportunity to use EHR data to identify eligible research cohorts and send recruitment invitations to potential participants via the patient portal [18,19]. Direct messaging through patient portals enables a study to efficiently contact eligible patients and facilitates low-touch, low-cost outreach to large numbers of patients, an approach that is particularly advantageous for studies with large target sample sizes. In addition, once a system of electronic recruitment is established, the process of identification, prescreening, and outreach can be automated and repeated.

Studies using patient portal research invitations for recruitment report a wide range of study enrollment rates, with 1.8% to 24.7% of those who received an invitation consenting to participate [20-27]. A summary of 14 studies that sent research recruitment invitations through the patient portal at a single medical center found that condition-specific studies had higher response and enrollment rates compared to general health studies [23]. The ADAPTABLE (Aspirin Dosing: A Patient-Centric Trial Assessing Benefits and Long Term Effectiveness) study, a pragmatic trial with a recruitment target of 15,000 participants, reported enrollment rates from four different modes of outreach: patient portal, email, mailed letter, and in-person communication with the research coordinator. Although in-person recruitment had the highest enrollment rates of all the modes, patient portal, and email outreach yielded the most overall study enrollment because they allowed the study team to approach many more potential participants than did the other modes [24]. A recent study comparing in-person, email, and patient portal recruitment of adults from primary care and bariatric clinics also found that electronic forms of outreach resulted in the most overall study participants in spite of lower recruitment efficiency compared to in-person recruitment [27].

Despite the advantages of using patient portals to recruit for research, they remain primarily clinical tools, and using them to send research invitations may run the risk of decreasing patients’ trust in the health care system or utilization of the platform for clinical purposes. However, there is some evidence that patients find recruitment through patient portals acceptable. Plante et al [25] reported only 2 complaints and 1 request to unsubscribe from future messages in a study that sent 6896 invitations, and Gleason et al [28] noted that most patients reported research recruitment to be an acceptable use of patient portals in a satisfaction survey from a study that sent 1303 invitations. Patients who find patient portal recruitment to be unacceptable, however, may not open a message, send a complaint, or complete a satisfaction survey. Thus an in-depth understanding of factors influencing acceptability of patient portal recruitment remains to be determined.

Demographic disparities between patient portal users and nonusers present a major barrier to the recruitment of a representative study sample. Studies have shown that patient portal nonusers are more likely to be racial/ethnic minorities, older, male, low socioeconomic status, low health literacy, and live in a rural area [8,23,29-31]. Patient portal recruitment may, however, decrease study population disparities that result from certain demographic groups being approached for research participation less frequently in clinic settings [32,33]. There is some evidence that clinicians as gatekeepers may contribute to the under-representation of certain populations, particularly among patients with minority backgrounds [33]. Mass electronic invitations may be a universal recruitment outreach approach reflective of demographic variation. Some studies have recommended that patient portal recruitment be one part of a comprehensive outreach approach, including approaches that specifically target traditionally under-represented groups [34].

### Early Check: A Research Study Piloting the use of a Patient Portal to Recruit Pregnant Women

In this article, we describe our use of invitations sent through the Epic EHR and patient portal (MyChart) within UNC Health (UNCH). At UNCH, the patient portal is branded *my* UNC *Chart*. We used *my* UNC *Chart* to recruit for Early Check, a research study offering screening to all newborns in the state of North Carolina for a panel of genetic conditions. With a target recruitment rate of over 10,000 newborns per year, an online consent process that does not require contact with a research coordinator, and broad eligibility criteria, Early Check is a study for which recruitment messaging through patient portals is a good fit. Additionally, the target populations for recruitment outreach are pregnant women and mothers of newborns; this group is relatively younger and female, both groups which have been shown to be more likely to open a patient portal account and use patient portals to manage their health [15]. Two recent studies reported a rate of patient portal utilization between 34% and 72% of pregnant patients [21,35]. One study recruited pregnant women to a research study through a patient portal and found 34% of pregnant patients used their patient portal, and when invited to their study, 11% consented and completed their questionnaire [21].

Since Early Check began recruitment in October 2018, the primary outreach method has been personalized direct mail letters and emails on letterhead from the North Carolina Department of Health and Human Services, a study partner, sent postnatally to all women with a listed mailing or email address in the North Carolina newborn screening records. A social media outreach campaign was piloted from March to September 2019. We resumed social media advertising on Facebook and Instagram on April 1, 2020. An evaluation of the direct mail outreach impact on study enrollment showed that approximately 4% of all women who were sent a recruitment letter enrolled their newborn in the study, and the enrollment rate among women who also received a recruitment email was approximately 5% [36]. An analysis of the social media campaign from March 2019 to September 2019 showed that paid ads on social media resulted in 3.5 additional daily enrollments in the study for each day ads were run [37]. To further increase outreach to eligible participants, we used *my* UNC *Chart* to send recruitment invitations to pregnant women whose newborns would be eligible for Early Check.

### Objective

In this article, we describe the use of a patient portal to recruit research participants for Early Check and report on characteristics of mothers who received and opened a research recruitment message and enrolled their newborns in the study. We addressed two research questions:

1. Is receipt of a research invitation through *my* UNC *Chart* patient portal associated with enrollment in the Early Check research study within 27 days after receipt of the invitation?
2. Is there a difference in opening a research invitation or enrollment in Early Check by a mother’s race/ethnicity, age, or rural/urban home address location?

To address these questions, we examined data on 4510 UNCH patients who were invited to participate in Early Check through *my* UNC *Chart* between November 22, 2019, and March 5, 2020.

## Methods

### Early Check Research Study

A collaboration between RTI International, the University of North Carolina at Chapel Hill, Wake Forest School of Medicine, Duke University, and the North Carolina State Laboratory of Public Health, Early Check is a large research study offering screening for a panel of conditions to all newborns in the state of North Carolina [38]. The panel includes fragile X syndrome (October 2018-current), spinal muscular atrophy (October 2018-March 2021), and Duchenne and related muscular dystrophies (November 2020-current). Newborns are eligible if they have a newborn screening in North Carolina and live in North Carolina or South Carolina. Newborns may be enrolled in the study by their mother or legally authorized representative in the event the mother is unavailable, between the start of the mother’s second trimester and when the newborn is one month old. During the phase of the study described herein, permission for the newborn to participate was completed entirely online without direct engagement with a research recruiter [39]. The Office of Human Research Ethics at the University of North Carolina at Chapel Hill serves as the central Institutional Review Board for Early Check (#18-0009), and they approved these activities.

### Recruitment Using my UNC Chart Invitations

The process of identifying women within UNCH to be sent an invitation to participate in Early Check via *my* UNC *Chart* began with a computable phenotype, a data query that “use[s] EHR data exclusively to describe clinical characteristics, events, and service patterns for a specific patient population [40].” UNCH’s enterprise data warehouse, the Carolina Data Warehouse for Health (CDWH), was queried using the computable phenotype to identify invitation recipients. UNCH’s appointment discharge paperwork provides patients with a unique ID that can be used to activate their *my* UNC *Chart* account. Women were invited if they had ever activated their *my* UNC *Chart* account, regardless of how recently they had logged into the account. The primary criteria in the computable phenotype were: a) having an active pregnancy “episode of care,” and b) being in the second or third trimester of pregnancy (ie, 13-42 weeks’ gestation).

In Epic@UNC, a pregnancy Episode of Care groups all prenatal encounters and diagnoses for a pregnancy. A pregnancy Episode of Care can be generated at any time during the pregnancy, although ideally, it is generated at the time a pregnancy is first confirmed or at the time a pregnant woman transfers her care from another health care system. Pregnancy Episodes of Care are designed to automatically resolve after delivery, specifically after any of the following: a) 48 weeks with no linked encounter; b) 364 days after the episode creation date; c) 84 days after the estimated delivery date in the patient’s medical record. An Episode of Care may also be manually resolved after the baby is born. Pregnancy Episodes of Care are not designed to resolve automatically when a woman loses her pregnancy; the Episode of Care must be manually resolved (eg, closed out) in the case of pregnancy loss.

Since a cohort based on active pregnancy Episodes of Care may unintentionally include some women who have lost their pregnancy, women were excluded from receiving an invitation if their health record showed any of a series of International Classification of Diseases 10th revision or current procedural terminology codes associated with elective or spontaneous abortion within 10 months of the date that the CDWH was queried. Women were also excluded if they had indicated in their communication preferences that UNCH was not permitted to contact them through *my* UNC *Chart*. Women were only sent one invitation, so they were also excluded from the cohort if they had already been sent an invitation for the study during the same pregnancy Episode of Care. The computable phenotype with inclusionary and exclusionary codes and a figure showing the text of the research invitation can be found in the Multimedia Appendices 1 and 2.

### Data

We consolidated data from four sources: (1) UNCH patient records with *my* UNC *Chart* invitation data; (2) ZIP code-level rural-urban commuting area (RUCA) approximation codes, from which we derived dichotomous urbanicity status; (3) newborn screening records gathered from the North Carolina State Laboratory of Public Health, to which Early Check mailing list data have been appended; and (4) enrollment information collected through the Early Check permission portal. After cleaning and standardizing the data, we iteratively matched records from *my* UNC *Chart* to the newborn screening and Early Check permission portal datasets using several combinations of data fields appearing in two or more sources, including phone number, email address, name, date of birth, and street address (including ZIP codes). We visually inspected the combined data set after each pass to find and update mismatched records or duplicates. The final data set used in the analysis contained one record per patient with variables derived from all four sources.

The main analyses presented in this report focus on all 4510 women living in North Carolina or South Carolina who had not yet enrolled in Early Check and were sent invitations via *my* UNC *Chart* from November 22, 2019, through March 5, 2020. To standardize results from batches of invitations sent on different dates, we set a 27-day window for recruitment and enrollment outcomes starting from the date participants were sent a *my* UNC *Chart* invitation. We selected a 27-day window to avoid any overlap with a social media ad campaign for Early Check that began April 1, 2020. We also compiled aggregate data for women with patient records in the UNCH system who did not have an active *my* UNC *Chart* account but would have otherwise met the eligibility criteria for an invitation. We used this aggregate data to estimate the proportion of Early Check-eligible UNCH patients who were reachable through *my* UNC *Chart* and to examine whether there are differences by age, race and ethnicity, or urbanicity between women who received an invitation and those who did not.

### Measures

#### Early Check Enrollment

We converted enrollment dates into a dichotomous variable indicating whether women granted permission for their babies to participate in Early Check within 27 days of being sent a *my* UNC *Chart* invitation*,* enrolled (1) or not enrolled (0). Among women who enrolled a child in the study within the 27-day timeframe, we also calculated the number of days it took them to enroll.

#### Opened my UNC Chart Invitation

Using the earliest date that women logged into their account to view the invitation, we derived a dichotomous variable recording whether women opened the invitation within 27 days of when it was sent, yes (1) or no (0). We also calculated the number of days it took women to open the invitation.

#### Contact Via Direct Mail and Email

Using variables recording the dates that postnatal Early Check outreach materials were sent, we created a set of dichotomous variables indicating whether each woman was sent a postnatal recruitment letter or email up to 27 days of being sent a *my* UNC *Chart* invitation (for each type of mailing, yes [1] or no [0]).

#### Age

We converted women’s date of birth to age in years, anchoring it to the date when we sent the recipient a *my* UNC *Chart* invitation (ie, invitation date-date of birth/365.25). We then transformed this into a 5-level categorical variable: Under 20, 20 to 24, 25 to 29, 30 to 34, and ≥35 years.

#### Race and Ethnicity

We used race and ethnicity data from the UNCH patient records and recoded these into a single variable that aligns with the race and ethnicity categories used in resident live birth reports published by the North Carolina Department of Health and Human Services: non-Hispanic White alone, non-Hispanic Black alone, Hispanic, and non-Hispanic any other race or unknown [41].

#### Urbanicity

To measure urbanicity, we constructed a variable based on RUCA codes associated with the patient residential ZIP codes recorded in the *my* UNC *Chart* data. RUCA codes were developed by the US Department of Agriculture to classify Census tracts by population density, proximity to large urban centers, and daily commuting flows [42]. For this analysis, we used ZIP code RUCA approximation codes developed by the University of Washington and recoded these into a two-level urbanicity measure developed for the National Cancer Institute’s Surveillance, Epidemiology, and End Results database [43,44]. Under this coding scheme, we collapsed RUCA codes associated with each ZIP code of residence into two categories indicating whether the location was urban area commuting focused (ie, urban) or not (ie, rural). ZIP codes with 1 of 10 RUCA codes (ie, 1.0, 1.1, 2.0, 2.1, 3.0, 4.1, 5.1, 7.1, 8.1, or 10.1) were classified as urban (1) and all other codes were classified as rural (0).

### Statistical Analysis

We conducted logistic regressions to test for differences in whether women enrolled their newborns in the study or opened the *my* UNC *Chart* invitation by outreach methods, urbanicity, race and ethnicity, and age. The model estimating enrollment included whether women opened the invitation as a predictor variable; all other regressors were the same in both models. Cases with missing values on one or more regressors were excluded by listwise deletion in the logistic regression models. In addition to reporting model estimates, we also present the predicted probabilities for significant categorical variables, which represent the rates of enrollment and invitation-opening within levels of those variables while controlling for other regressors in the models. To examine potential differences by urbanicity, race and ethnicity, and age between women who were sent invitations through *my* UNC *Chart* and patients in the UNCH system who were otherwise eligible but did not have an active *my* UNC *Chart* account, we conducted *χ*^2^ tests of independence. We followed up significant *χ*^2^ tests involving independent variables with more than 2 categories using two-sample *z* tests for the difference of proportions. For these pairwise comparisons, we used a Bonferroni-adjusted alpha level of .0085. We conducted all analyses using Stata Statistical Software (version16.0; StataCorp).

## Results

### Sample Characteristics

In total, 12,036 patients within the UNCH system fit the computable phenotype that would have made them eligible to receive an invitation from November 22, 2019, through March 5, 2020, but only 4510 out of 12,036 (37.5%) had an active *my* UNC *Chart* patient portal account. We compared the demographic characteristics of women to whom we sent a *my* UNC *Chart* invitation to those who did not receive an invitation because they did not have an active account. We found no significant differences by age (*χ*^2^ [4, *N*=12,036]=3.51; *P*=.48) or urbanicity (*χ*^2^[2, *N*=12,036]=0.37; *P*=.54), but we did find differences by race/ethnicity (*χ*^2^[3, *N* = 12,036] = 180.99; *P*<.001, Cramér’s V = 0.12). A greater percentage of non-Hispanic White patients (2527/5852, 43.2%) had an active *my* UNC *Chart* account compared to non-Hispanic Black patients (932/2738, 34.0%, *z*=8.05; *P*<.001), Hispanic patients (531/1,916, 27.7%, *z*=12.03; *P*<.001), or non-Hispanic patients of any other race (520/1530, 34.0%, *z*=6.50; *P*<.001). Hispanic patients were significantly less likely to have an active *my* UNC *Chart* account than patients in any of the other three race/ethnicity groups. The full contingency table comparing whether women had an active *my* UNC *Chart* account by race and ethnicity is shown in Table 1.

Characteristics of North Carolina or South Carolina residents who were sent an invitation to participate in Early Check from November 22, 2019, through March 5, 2020, are presented in Table 2. The sample excludes 18 patients who had already enrolled their newborns in Early Check before their *my* UNC *Chart* invitation was sent. Invitations were sent in five batches, with approximately half of the recipients (2466/4510, 54.7%) included in the first mailing on November 22, 2019. Two-thirds of all recipients (3054/4510, 67.7%) logged into their *my* UNC *Chart* accounts and opened the invitation within 27 days of when it was sent. Among women who opened the invitation, it took them 2.45 days (SD 4.83) on average to do so; however, the distribution is positively skewed, with 68.6% (2094/4510) opening it within 24 hours. Relatively few women (357/4510, 7.9%) were sent a postnatal recruitment letter or a personalized email (24/4510, 0.5%) within 27 days of being sent the *my* UNC *Chart* invitation. This observation is not unexpected, given that the computable phenotype was designed to target women with an active pregnancy as early as the 13th week of gestation.

**Table 1:** Cross-tabulation of having an active *my* UNC *Chart* account by race and ethnicity (N=12,036).^a^

Active *my* UNC *Chart* account	Race/ethnicity, n(%)				
	White, n	Black	Hispanic	Other	Total
Yes	2527 (43.2)	932 (34.0^b^)	531 (27.7^c^)	520 (34.0^b^)	4510 (37.5)
No	3325 (56.8)	1806 (66.0)	1385 (72.3)	1010 (66.0)	7526 (62.5)
Total	5852 (100.0)	2738 (100.0)	1916 (100.0)	1530 (100.0)	12,036 (100.0)

^a^*χ*^2^(3,*N*=12036)=180.99; *P*<.001, Cramér’s V= 0.12

^b, c^Percentages among participants with an active *my* UNC *Chart*account across race/ethnicity columns are significantly different at a Bonferroni-adjusted *P*<.009

**Table 2:** Characteristics of women who were sent a *my* UNC *Chart* invitation (N=4510).^a^

Characteristic				Values, n (%)^b^
**Enrolled in Early Check within 27 days of *my* UNC *Chart* invitation^c^**				
	Yes		161 (3.6)	
	No		4349 (96.4)	
**Opened *my* UNC *Chart* invitation within 27 days**				
	Yes		3054 (67.7)	
	No		1456 (32.3)	
**Postnatal outreach methods sent within 27 days of *my* UNC *Chart* invitation**				
	**Recruitment letter**			
		Sent letter	357 (7.9)	
		No letter	4153 (92.1)	
	**Personalized email**			
		Sent email	24 (0.5)	
		No email	4486 (99.5)	
**Date invitation sent**				
	November 22, 2019		2466 (54.7)	
	January 7, 2020		931 (20.6)	
	January 29, 2020		423 (9.4)	
	February 12, 2020		272 (6.0)	
	March 5, 2020		418 (9.3)	
**Age (years)**				
	Under 20		169 (3.7)	
	20-24		791 (17.5)	
	25-29		1224 (27.1)	
	30-34		1386 (30.7)	
	≥35		940 (20.8)	
**Race/ethnicity**				
	White		2527 (56.0)	
	Black		932 (20.7)	
	Hispanic		531 (11.8)	
	Other or unknown		520 (11.5)	
**Urbanicity**				
	Urban		3615 (80.2)	
	Rural		892 (19.8)	
	Unknown		3 (0.1)	

^a^Analysis excludes 18 patients who were sent a *my* UNC *Chart* invitation after enrolling in Early Check.

^b^Percentages may not sum to 100 due to rounding.

^c^For this analysis, we set a 27-day enrollment window from the date the *my* UNC *Chart* invitations were sent to normalize results from batches of invitations sent on different dates.

### Early Check Enrollment

In all, 3.6% (161/4510) of women who received a *my* UNC *Chart* invitation enrolled their newborns in the study within 27 days. Excluding 8 women who enrolled their newborns in Early Check without opening the invitation, women took on average 3.92 days (SD 6.50) to enroll. Similar to the distribution for the time it took to open the invitation, the enrollment timing distribution was positively skewed, with 63.4% (97/4510) enrolling within 1 day of opening the invitation.

Our first research question examined whether and to what extent women who opened a research invitation sent to them through *my* UNC *Chart* were more likely to enroll in Early Check within 27 days of receiving the invitation. The overall logistic regression model predicting enrollment was significant (*χ*^2^ [11, *N*=4507]=134.90; *P*<.001, *R^2^*_McFadden_=.10). As shown in Table 3, the odds of enrolling among women who opened the invitation was nearly 9 times the odds of enrolling among women who did not open and who therefore did not view their invitation (SE 3.24, OR 8.86, 95% CI 4.33-18.13; *P*<.001). Expressed in terms of predicted probabilities holding everything else in the model constant, 4.88% of women who opened the invitation (SE 0.38 , 95% CI 4.13%-5.63%) enrolled their newborns in Early Check within 27 days of when it was sent compared to only 0.58% of women who did not open the invitation within that time frame (SE 0.02, 95% CI 0.18%-0.99%) and most likely became aware of the study through another outreach method Being sent a postnatal recruitment letter (*P*=.57 or a personalized email invitation (*P*=.53) did not have a significant additional impact on enrollment within the 27-day period.

Our second research question asked, in part, whether there are differences in enrollment by race/ethnicity, age, or urbanicity. Although we observed no significant differences in enrollment rates across age groups, race/ethnicity and urbanicity were both related to enrollment. The odds of enrolling for Black women who were sent a *my* UNC *Chart* invitation was 0.29 times the odds of White women (SE 0.09, OR 0.29, 95% CI 0.16-0.55; *P*<.001). Expressed in terms of predicted probabilities, whereas 4.49% of White women (SE 0.40, 95% CI 3.72%-5.27%) enrolled their newborns in Early Check within 27 days of when their invitations were sent, only 1.38% of Black women enrolled their newborns (SE 0.41, 95% CI 0.57%-2.19%). We found no other differences in enrollment by other race/ethnicity groups. Additionally, women with a home address in urban zip codes were more likely to enroll than women from rural zip codes (SE 0.97, OR 3.03, 95% CI 1.62-5.67; *P*=.001). Controlling for the other variables in the model and expressed in terms of predicted probabilities, 4.04% of urban women (SE 0.32, 95% CI 3.41%-4.67%) enrolled their newborns in Early Check compared to 1.40% of rural women (SE 0.42, 95% CI 0.57%-2.22%).

**Table 3:** Logistic regression analysis predicting Early Check enrollment (*N*= 4507).

Predictor		OR	SE	*P* values	95% CI^a^
**Opened invitation within 27 days of when it was sent**					
	Reference = no	1.0	—^b^	—	—
	Yes	8.86	3.24	<.001	4.33-18.13
**Postnatal recruitment letter**					
	Reference = not sent a recruitment letter	1.0	—	—	—
	Sent a recruitment letter	1.20	0.37	.565	0.65-2.20
**Postnatal personalized email invitation**					
	Reference = not sent an email invitation	1.0	—	—	—
	Sent an email invitation	1.98	2.16	.531	0.23-16.82
**Race/ethnicity**					
	Reference = White	1.0	—	—	—
	Black	0.29	0.09	<.001	0.16-0.55
	Hispanic	0.63	0.20	.135	0.34-1.16
	Other	0.62	0.16	.068	0.36-1.04
**Urbanicity^c^**					
	Reference = rural	1.0	—	—	—
	Urban	3.03	0.97	.001	1.62-5.67
**Age (years)**					
	Reference = under 20	1.0	—	—	—
	20-24	0.97	0.75	.969	0.21-4.43
	25-29	1.87	1.38	.396	0.44-7.92
	30-34	1.92	1.41	.376	0.45-8.07
	≥35	2.58	1.90	.198	0.61-10.90
Constant		0.00	0.00	<.001	0.00-0.01

^a^The analysis excluded 3 women for whom geolocation data were insufficient to compute urbanicity.

^b^The reference levels are fixed parameters, not estimates, so no measures of precision were calculated.

^c^'Urbanicity' is a variable indicating whether women live in an urban or rural area based on residential ZIP code (see measures section).

### Opened my UNC Chart Invitation

Our second research question also considers associations of race/ethnicity, age, and urbanicity on whether women opened the research invitation sent to them through *my* UNC *Chart*. As shown in Table 4, the logistic regression model that predicts opening the invitation was significant (*χ*^2^[10, *N*=4507]=62.38; *P*<.001, *R^2^*_McFadden_=.01). Women who were sent a postnatal recruitment letter by mail within 27 days of a *my* UNC *Chart* invitation were significantly less likely to open the invitation (SE 0.09, OR 0.76, 95% CI 0.60-0.96; *P*=.02). Holding everything else constant and expressed in terms of predicted probabilities, 62.1% of women who were sent a postnatal recruitment letter opened their *my* UNC *Chart* invitations (SE 2.6, 95% CI 56.9%-67.2%) versus 68.2% of women who were not sent a recruitment letter (SE 0.7, 95% CI 66.8%-69.6%). Whether women were sent a postnatal personalized email within the 27-day timeframe was not significantly associated with opening the *my* UNC *Chart* invitation (*P*=.19), nor was urbanicity (*P*=.75). However, race/ethnicity and age were both significantly related to opening the invitation. Black women were significantly less likely than White women to open the invitation (SE 0.05, OR 0.67, 95% CI 0.57-0.78; *P*<.001), with 61.4% of Black women (SE 1.6, 95% CI 58.3%-64.5%) opening the invitation compared to 70.4% of White women (SE 0.9, 95% CI 68.6%-72.2%). Hispanic women were also less likely to open the invitation than were White women (SE 0.07, OR 0.73, 95% CI 0.60-0.89; *P*=.002), with an estimated 63.4% of Hispanic women opening their invitations. Lastly, opening the *my* UNC *Chart* invitation differed significantly by age. Compared to women under 20 years of age, women aged 25 to 29 years (SE 0.26, OR 1.51, 95% CI 1.08-2.10; *P*=.02), 30 to 34 years (SE 0.28, OR 1.67, 95% CI 1.20-2.33; *P*=.003), or 35 years or more (SE 0.25, OR 1.44, 95% CI 1.03-2.03; *P*=.35) were significantly more likely to open the invitation. The predicted probabilities of opening the *my* UNC *Chart* invitation by age are shown in Table 5.

**Table 4:** Logistic regression analysis predicting whether the *my* UNC *Chart* invitation was opened (*N*=4507).

Predictor		OR	SE	*P* values	95% CI
**Postnatal recruitment letter**					
	Reference = not sent a recruitment letter	1.0	—^a^	—	—
	Sent a recruitment letter	0.76	0.09	.022	0.60-0.96
**Postnatal personalized email invitation**					
	Reference = not sent an email invitation	1.0	—	—	—
	Sent an email invitation	0.57	0.24	.185	0.24-1.31
**Race/ethnicity**					
	Reference = White	1.0	—	—	—
	Black	0.67	0.05	< .001	0.57-0.78
	Hispanic	0.73	0.07	.002	0.60-0.89
	Other	0.99	0.11	.928	0.80-1.22
**Urbanicity^b^**					
	Reference = rural	1.0	—	—	—
	Urban	0.97	0.08	.747	0.83-1.14
**Age, years**					
	Reference = under 20	1.0	—	—	—
	20-24	1.26	0.22	.178	0.90-1.78
	25-29	1.51	0.26	.015	1.08-2.10
	30-34	1.67	0.28	.003	1.20-2.33
	≥35	1.44	0.25	.035	1.03-2.03
**Constant**		1.70	0.29	.002	1.22-2.38

^a^The reference levels are fixed parameters, not estimates, so no measures of precision were calculated.

^b^The analysis excluded 3 women for whom geolocation data were insufficient to compute urbanicity.

**Table 5:** Predicted probability of opening a *my* UNC *Chart* invitation by age (*N*= 4507).

Predictor		%^a^	SE	95% CI
**Age, years**				
	Under 20	58.9	3.8	51.5-66.3
	20-24	64.4	1.7	61.0-67.7
	25-29	68.3	1.3	65.7-70.9
	30-34	70.4	1.2	68.0-72.8
	≥35	67.3	1.5	64.3-70.4

^a^Predicted probability expressed as a percentage controlling for covariates included in the logistic regression model.

## Discussion

### Principal Findings

We examined the utility of sending research invitations to pregnant women through a patient portal and whether opening an invitation was associated with enrollment in the study. We found an association between opening a patient portal research invitation and enrollment in the study but found disparities by race/ethnicity in having a *my* UNC *Chart* patient portal, opening the invitation, and enrolling in the study.

The use of EHR data to identify and contact eligible participants through their patient portal proved to be successful. The findings show that the *my* UNC *Chart* patient portal within UNCH could be used to send recruitment invitations to over 4500 pregnant women whose newborns would be eligible for Early Check over a period of approximately 15 weeks. These results demonstrate the efficiency of using patient portals to send recruitment invitations to large numbers of potential research participants, compared to the time and effort it would require to contact thousands of participants through other methods like phone or in-person recruitment. As such, patient portals are especially valuable for studies seeking to approach and enroll very large numbers of participants.

Despite contacting thousands of eligible women, those we contacted accounted for a minority (4510/12.036, 37.5%) of patients at UNCH who met the computable phenotype during the time period of this study; a majority of eligible women did not have active *my* UNC *Chart* accounts and thus could not receive a recruitment message. However, for a study like Early Check with broad eligibility criteria and for which patients will become newly eligible over time as women become pregnant, *my* UNC *Chart* still proved an efficient method to contact thousands of eligible women.

Overall, patient portal research invitations sent through *my* UNC *Chart* were associated with enrollment in the Early Check study among women who opened those invitations. We found that a majority of women who received a *my* UNC *Chart* research invitation opened it, and of those who opened it, 4.88%, expressed in predicted probability, enrolled their newborn. In comparison, a previous analysis of the other primary recruitment method for Early Check, postnatal letters and emails to new mothers showed an overall statewide enrollment rate of 4% [36]. For those women who were sent a postnatal letter in addition to the recruitment invitation through *my* UNC *Chart*, the receipt of the postnatal letter did not increase the odds of enrollment. We did not independently compare *my* UNC *Chart* recruitment with direct letters and emails.

The findings demonstrated disparities in the use of patient portals, opening of research invitations, and enrollment in the study by race/ethnicity. There were also disparities in enrollment by urban/rural home address and in opening research invitations by age. Black women and Hispanic women were less likely to open an Early Check recruitment invitation sent through *my* UNC *Chart* and were less likely to enroll in the study after opening the invitation compared to non-Hispanic White women. We also found disparities by race and ethnicity among women we had hoped to reach using *my* UNC *Chart* invitations because they did not have an active *my* UNC *Chart* account. Members of traditionally underrepresented racial and ethnic minority groups were less likely than non-Hispanic White women in our target audience to have an active *my* UNC *Chart* account. Hispanic women were least likely to be *my* UNC *Chart* users, a finding that may be partially due to the availability of *my* UNC *Chart* in English only.

In our analysis of *my* UNC *Chart* by age and rural/urban home address, we found that there was no difference by age or urbanicity in those who had an active *my* UNC *Chart* account. Age was not significantly associated with opening the message or enrolling in the study except for women less than 20 years of age who were less likely to open the invitation. We found that women from urban areas were significantly more likely to enroll their newborns in the study compared to women from rural areas. It is not clear why urban women were more likely to enroll their newborns in the study although proximity to academic medical institutions and research familiarity may play a role.

### Comparison With Prior Work

Our enrollment rate among women who received Early Check *my* UNC *Chart* patient portal research recruitment invitations was similar to other studies using patient portals for recruitment, including the ADAPTABLE study performed in the same health system using a similar messaging protocol (4.4%) and a review of 13 studies recruiting through the patient portal of a single health system (2.9%-3.4%) [20,23,24]. Some studies have reported higher enrollment rates using patient portals ranging from 7% to 38% [22,28,32,34]. Bower et al [21], a study that also recruited pregnant women through patient portals, had a higher enrollment rate (11%) compared to the enrollment rate we report here (161/4510, 3.6%). The reasons for the differing enrollment rates across these studies are unclear but may be partially due to the target study population, demographics of patient portal users at an institution, type of study, demand on participants, formatting of the message, and the timing of the invitation in relation to a scheduled medical appointment. More research is needed on the factors associated with successful recruitment through patient portals and on the acceptability of using patient portals to recruit for research, to identify those studies for which a patient portal recruitment approach is likely to be most productive and acceptable.

The findings of racial and ethnic disparities in the users of *my* UNC *Chart*, opening of the recruitment invitation, and enrollment in the study are consistent with the findings across other studies examining the use of patient portals for recruitment and the use of patient portals for clinical care [8,23,29,32,34]. It is important to recognize that patient portal recruitment approaches have limited reach and may compound the problem of underrepresentation in health research. Identifying barriers to patient portal use for clinical care and intervening with specific subgroups to address those barriers may improve the reach of patient portals and their utility in recruiting a diverse research sample [17]. In the meantime, research administrators should use patient portals as part of a broader recruitment strategy and not the sole recruitment method.

### Limitations

The study examined patient portal research invitations sent to pregnant women, and findings may have limited generalizability to other types of patients. Findings may also have limited generalizability to organizations that use a patient portal other than Epic MyChart. It is also a limitation of the study that we did not directly compare the effectiveness of recruitment to Early Check through *my* UNC *Chart* research invitations to recruitment through postnatal letters and emails. We were unable to conclude whether one of these recruitment approaches was superior in enrolling newborns in the Early Check study or whether one approach would have resulted in a more representative sample.

### Conclusions

Patient portals are an effective way to recruit participants for research studies and are especially useful for studies with large target sample sizes. There remain substantial racial and ethnic disparities in the use of patient portals, the response to receipt of an invitation, and enrollment in the study.

## Appendix

Multimedia Appendix 1 Computable Phenotype for Carolina Data Warehouse for Health Query (CDWH).

Multimedia Appendix 2 Text of the Early Check *my* UNC *Chart* Research Invitation.

## Abbreviations

CDWH: Carolina Data Warehouse for Health
EHR: electronic health record
NCATS: National Center for Advancing Translational Sciences
RUCA: rural-urban commuting area
UNCH: UNC Health
