# Supplementary material for: Using a Patient Portal to Increase Enrollment in a Newborn Screening Research Study: Observational Study
Source: JMIR Pediatr Parent. 2022 Feb 10;5(1):e30941. doi: 10.2196/30941 (PMC8874929; doi:10.2196/30941)
Supplement: Multimedia Appendix 1 [file pediatrics_v5i1e30941_app1.pdf]

Criteria	Inclusionary or Exclusionary	Codes[a]
Active Pregnancy	Inclusion	-
Gestational Age (13w0d – 42w0d)	Inclusion	-
CPT Codes related to abortion or miscarriages	Exclusion	59820, 59821, 59812, 58120
Diagnosis Codes related to abortion or miscarriages	Exclusion	O03%, O02.1%, Z33.2%
Diagnosis Procedures Codes related to abortion or miscarriages	Exclusion	10D1%, 10A%

[a] % represents wildcard, and any sub-code of that code family. The CDWH was queried every two weeks to identify and send messages to a new cohort of recipients. Invitations were sent via Epic's bulk messaging feature. The computable phenotype was refined between the first and second batch of invitations. Figure 1 shows the text of the research invitation. Most participants received an email from UNCH alerting them that they had a new message in *my* UNC *Chart*. Invited women could choose whether to log into their *my* UNC *Chart* account to view the invitation which included basic information about the study, including: the study PI, a brief description of how Early Check works, the conditions included in Early Check's screening panel, and a hyperlink to Early Check's secure online consent portal. Because Early Check consents occur via the secure online portal, and women provide contact information and other identifiable information at the time of consent, there is no need for additional tracking via Epic (e.g., for the purpose of follow-up by a study coordinator) once the invitation is sent.

[ ] This is a Multimedia Appendix to a full manuscript published in the J Med Internet Res. For full copyright and citation information see http://dx.doi.org/10.2196/30941
