# Supplementary material for: Using a Patient Portal to Increase Enrollment in a Newborn Screening Research Study: Observational Study
Source: JMIR Pediatr Parent. 2022 Feb 10;5(1):e30941. doi: 10.2196/30941 (PMC8874929; doi:10.2196/30941)
Supplement: Multimedia Appendix 2 [file pediatrics_v5i1e30941_app2.pdf]

Research Opportunity: Early Check

Early Check: A study testing babies for rare health problems

Principal Investigator

Cynthia M. Powell, MD, MS

Overview

- We are inviting you to take part in Early Check, a research study.
- Early Check offers free tests to check your baby for rare health problems. Right now Early Check is testing for spinal muscular atrophy (SMA) and fragile X syndrome.
- Early Check is a partnership between UNC-Chapel Hill, Duke University, Wake Forest School of Medicine, the North Carolina State Laboratory for Public Health, and RTI International.
- Sign up and learn more at portal.EarlyCheck.org.

Description

We are inviting all women who recently visited a UNC Health Care OB clinic to take part in Early Check. Early Check is a research study that offers babies free tests for rare health problems that are not part of regular newborn screening in NC.

In NC, all babies have a heel prick in the hospital for regular newborn screening. Early Check uses those same drops of blood to do extra screening tests. There's nothing else that Early Check needs from you or your baby, but you do need to sign up online first.

Right now, Early Check is testing for spinal muscular atrophy (SMA) and fragile X syndrome. SMA causes muscle weakness that gets worse over time. There is a new treatment and ongoing research, but SMA could still cause early death for some babies. Fragile X syndrome causes learning and behavior problems. Fragile X syndrome does not cause early death. Because Early Check is a research study, the conditions that it tests for may change over time.

Early Check tests for ***rare*** health problems, so most parents get a normal result for their baby. If the Early Check tests find that your baby has SMA or fragile X syndrome, you will receive confirmatory testing and genetic counseling at no cost. We will also help you to find specialty medical care for your baby.

You can join Early Check if you're more than 12 weeks pregnant, or if you have recently had a baby who was born in NC. But you must sign up online before your baby is 4 weeks old.

Learn more

To learn more about Early Check and decide if you want to sign up, visit Early Check's secure website: portal.EarlyCheck.org.

[ ] This is a Multimedia Appendix to a full manuscript published in the J Med Internet Res. For full copyright and citation information see http://dx.doi.org/10.2196/30941
